# Supplementary material for: Are carbon emissions trading and green financial instruments synergistic? -Comprehensive quantitative research based on content analysis
Source: PLoS One. 2024 Mar 7;19(3):e0298601. doi: 10.1371/journal.pone.0298601 (PMC10919601; doi:10.1371/journal.pone.0298601)
Supplement: S1 Data — (ZIP) [file pone.0298601.s001.zip › Data/地级市Z_F工作报告.zip!/╔╜╬≈/╠1⁄2╘¡╩╨/└┤╫╘╣1⁄2╓┌║┼í╛┬φ┐╦╩2╛▌═°í┐_2010.docx]

各位代表：

现在，我代表市政府向大会报告工作，请予审议，并请各位政协委员和列席人员提出意见。

一、2009年政府工作回顾

2009年，受国际金融危机影响，我市经济发展遇到了严峻挑战。我们在上级和市委的正确领导下，以科学发展观为指导，围绕“保增长、保民生、保稳定、促转型”，采取措施，积极应对，经济回升向好，社会事业全面进步。完成地区生产总值1545.2亿元，增长2.6%；全社会固定资产投资782亿元，增长11.3%；社会消费品零售总额721.7亿元，增长16.4%；城市居民人均可支配收入15607元，增长2.5%；农民人均纯收入6828元，增长7.4%；规模以上工业增加值470.1亿元，下降9.9%；财政总收入279.6亿元，下降8.9%；一般预算收入117.5亿元，增长0.5%；居民消费价格总水平下降0.1%。向全市人民承诺的十件实事全部完成！

（一）产业结构调整加快，“保增长、促转型”年度任务基本完成。

深入调整工业结构，重点项目加速推进。积极构建绿色工业体系，加快特色工业园项目建设，铁路装备工业园、太重煤机二期等一批重大项目开工建设。长安重汽3万辆重型汽车、太钢5万吨不锈钢管等一批项目建成投产或部分投产。15个项目列入省技术改造扶持项目。晋机火车轴、汾机风力发电等一批有核心竞争力的产品成为拉动我市工业增长的重要力量。加快煤炭资源整合和煤矿兼并重组步伐，煤矿数量压减60%，总产能提高24.3%，装备、技术、管理水平全面升级。规模以上工业企业万元增加值能耗下降7.2%。民营经济增加值完成700亿元，增长3%。高新区、经济区、民营区工业总产值分别增长8%、10%、 8%。

积极构建绿色农业体系，农村经济社会健康发展。农村经济总收入591亿元，增长7%。加快发展绿色农业项目，引进推广7大类蔬菜新品种，建成万间温室群无公害蔬菜生产基地5个。产业化经营稳步发展，组建了以水塔、蒙牛、森泰、河西市场为龙头的醋业、乳业、畜牧业和农产品加工流通集团。农业产业化企业实现销售收入60亿元。农民专业合作社发展到1507个，增速创全省第一。农业实用技术带动10万农户致富。推广可再生能源建设，建成农村大中型沼气和秸秆气化集中供气工程5座。解决11.4万农村人口的饮水安全问题。新改建农村公路515公里，完成村村通水泥（油）路236公里、通达工程121.8公里。转移农村富余劳动力2.1万人。2.4万农民脱贫。

城乡消费不断扩大，第三产业强劲增长。第三产业投资增长57%，完成增加值838.6亿元，增长10.2%，占GDP比重达到54.3%，拉动GDP增长5.4个百分点。蓝海汽配城、滨西商务中心等项目建成投运。6个农产品批发市场和10个农产品流通企业列入全省“双三十市场工程”项目。新建、改建农家店326家，已覆盖全市90%以上的行政村。组织开展“家电下乡”、“农机下乡”，有力拉动农村消费。实施旅游“一卡通”、“一证通”等促销活动，被国务院批准成为第二批居民对台旅游城市。实现旅游总收入195.2亿元，增长17.8%。积极开拓海外市场，新增外贸企业76家。推出现代服务业优质项目群，利用外资结构进一步优化，实际到位外资2.62亿美元，到位内资107.9亿元。

（二）重大项目群建设陆续展开，城乡一体化规划建设拉开框架。

八大功能区建设重点展开。按照城乡一体化建设发展的思路，将148项建设项目纳入八大功能区建设考核范围。目前，已竣工项目52个，在建项目55个，完成投资184.5亿元。

西山地区综合治理全面启动。编制完成西山整治区域的总体规划和8.4平方公里启动区的控制性详细规划。生态治理和恢复加快推进，“一湖六河”治理等工程陆续启动，89家产能落后企业已经关停，76座小煤矿已经关闭。企业搬迁工作扎实推进。新产业发展迈出可喜步伐，铁路装备园奠基开工，投资60亿元的北车集团项目加紧建设。基础设施建设全面展开，长风西大街、太古高速路、西山货运专线、旅游循环公路开工建设。

城乡规划与建设进展顺利。狠抓规划覆盖工程，完成城市总体规划与土地利用规划的对接和统一；太原经济圈战略规划编制完成；历史文化名城保护规划、轨道交通网线规划等重点规划编制工作取得重要进展；完成56个片区的规划编制工作，主城区控规全覆盖目标基本实现。实施城市建设投资超百亿、项目超百个的“双百工程”，共开工重点建设项目101项，改造小街小巷76条，完成投资117.3亿元。其中，实施城市主次干道工程28项、桥梁工程5项，迎泽西大街微循环、漪汾桥改造等工程全面完工；南内环桥、桃园南北路改造等工程主路完工通车；祥云桥、五龙口街改造等工程顺利推进。实施城市配套工程41项，部分工程建成投用。长风商务区等片区和小村镇加快建设。石太高速客运铁路等国家和省在我市的重点建设项目进展顺利。实施7项防洪治污工程、4项节能减排工程。房地产开发投资165亿元，增长35.7%；廉租房、经济适用房建设和棚户区改造开复工193万平方米，完成年度任务。城中村改造拆除42万平方米。建筑领域绿色转型取得重要进展，建筑节能改造82.5万平方米，获得第一批“国家可再生能源建筑应用示范城市”荣誉，争取到国家每年1.6亿元的资金支持。

环境治理和生态建设取得重要突破。严守“绿色高压线”，关停、淘汰污染企业及落后生产设施96家（座），完成一批重点污染源治理工程。拆除城中村土小锅炉8202台。机动车尾气达标率85.1%。对汾河流域三公里范围内的94家企业实施废水深度处理。组织开展饮用水源地保护、保障国庆环境质量等六大环保专项执法行动。圆满完成省下达的减排目标，市区二级以上天数296天。植树造林25.2万亩。实施园林绿化工程55项，汾河治理美化南延至长风桥段、学府公园等工程全面完工；汾河治理美化北延、环城高速互通绿化改造等工程基本完工。老龙头和蒙山两个地质环境治理项目获得国家资金支持。创建园林绿化单位32个，建成2个省级环境优美乡镇、20个省级生态文明村。全市绿化覆盖率达到35.78%，绿地率30.98%，人均公共绿地9.09平方米，被评为“国家园林城市”，实现了创建工作的历史性突破。

城市管理服务水平持续提高。深入推进 “城乡清洁工程”，积极开展“四治、五进、十创”活动，垃圾处理达到日产日清无积存要求，城乡环境明显改善，治超工作实现“四个零”目标。加强对建筑及房地产市场的监管，建设领域专项治理与容积率专项治理成效明显。稳步实施数字城管，通过国家住建部专家组验收。国土资源基础测绘工作在全国首家荣获“全国数字城市示范市”称号。集中供热扩网979万平方米。天然气置换10.3万户。12319城建服务热线事项办结率99.4%，市民满意率95.7%。

（三）千方百计“保民生、保稳定”，和谐社会建设取得新成绩。

就业和社会保障工作稳步推进。积极开展创业型城市创建工作，全市城镇新增就业11.8万人，下岗失业人员再就业4.4万人，创业就业2.4万人，城镇登记失业率为3.41%。五大社会保险参保人数均创历史新高。积极开展新型农村社会养老保险试点工作，建立被征地农民养老保险制度。全国劳动保障“两网化”管理试点工作扎实推进。普惠型社会救助体系建设进一步完善，6.4万农村困难群众纳入低保，解决1万余名重点优抚对象医疗难问题。在全国省会城市率先实施社会散居孤儿最低养育制度。慈善事业救助贫困人群10.2万人次，市慈善总会获得中华慈善突出贡献奖。

有效解决一批涉及群众利益的热点问题。加强价格监测预警，重要的居民生活必需品价格保持稳定。为3.5万户城乡低保户及低保边缘户发放供热券3000万元。新增公交车333辆，市民出行条件得到改善。建成162家放心粮油店。认真开展“清费减负促进发展年”活动，全年减负2亿元。开展食品药品安全专项整治，严把食品、药品市场准入关、检测关、退市关。建立完善重点流通企业、重要生产资料市场、肉食市场、成品油市场、酒类市场、农村市场、特殊行业等市场监管体系，确保市场供应和安全。12345便民服务热线受理来电74.8万个，办结率99.5%，满意率92.1%。

特色文化名城建设取得新成果。大力推进文化产业发展，在全省率先完成文化体制改革任务。经营性文化事业单位转企改制步伐加快，组建龙城电影集团、文广集团、高新区创意产业集团、报业集团、新晋商联盟五大文化产业集团。加强文化基础设施建设，太原美术馆、湖滨广场等项目顺利开工。积极开展申报国家级历史文化名城的工作，太原博物馆、晋阳古城国家考古遗址公园等建设项目加快推进。完成第三次全国文物普查。成功举办纪念新中国成立60周年和太原解放60周年系列文化活动。非物质文化遗产保护工作成效明显，共建名录141项，13项列入国家级名录。

各项社会事业稳步发展。强力推进“百校兴学”工程、校舍安全工程、校校有标准操场工程，各类项目学校竣工198所，在建69所，新增学位2.2万个。消除危房52.5万平方米，校舍安全工程太原模式在全国推广。8个县区通过省义务教育标准化验收。继续实行“两免一补”，45万名学生受益。开展全民终身学习，获得“全国全民终身学习活动先进城市”荣誉。太原大学新校区开工建设。启动公立医院改革试点。改扩建18所乡镇卫生院，县乡两级医疗卫生机构基础设施达标率达到81.7%。全市农村新型合作医疗参合率和人均筹资标准继续保持全省领先。有效防控甲型H1N1流感疫情。群众体育、竞技体育蓬勃开展，兴建全民健身路径和农民健身工程千余条（块）。滨河体育场建设进展顺利。圆满完成太原市第二次全国经济普查任务，荣获国家级先进。杏花岭区被评为全国和谐社区建设示范城区。气象、地震、计生、外事、侨台、档案、地方志、民防、双拥、国防后备力量建设等工作和老龄、妇女、残疾人、红十字、关心下一代、革命老区建设等事业都取得了新成绩。

社会形势和谐稳定。成功完成“长城6号”国家反恐怖实战演练。深入开展“晋阳风暴”二号、三号专项行动，圆满完成建国60周年庆典安保任务。“两抢一盗”警情下降19.6%。大力开展“整治酒后驾驶”等交通安全集中整治行动。深入开展“安全生产年”活动，全面落实企业安全生产、政府安全监管两个主体责任，在全市各行业各领域实施为期一年的安全生产专项整治，狠抓煤矿兼并重组、资源整合和尾矿库关闭工作，强化安全生产监管执法，严厉打击非法违法生产经营行为，全年未发生重大安全生产事故，安全生产形势保持稳定。信访形势明显好转，初信初访办结率达85%，成功处置大规模群体访169批，进京非正常访、赴省集体访、越级重复访分别下降28.5%、15.5%和20.4%。成功调解各类矛盾纠纷2.6万余起。建立四级自然灾害应急机制，成功避让4起地质灾害，积极应对百年一遇的暴雪灾害，防灾减灾扎实有效。

（四）围绕转型发展主要目标，体制机制创新迈出新步伐。

绿色转型推进机制进一步强化。出台《太原市绿色转型促进条例实施办法》，《太原市绿色转型促进条例》正式实施，我市成为全国第一个以地方立法形式整体推动绿色转型和区域科学发展的城市。绿色转型重点标准体系不断完善，制定和发布《太原市绿色村庄要求与评价》等绿色标准7个，总数达到27个，我市成为拥有绿色地方标准最多的城市。在机关、企业、农村等12个领域500家单位延伸和拓展“绿色十佳”创建活动。制定出台《建立健全绿色和循环经济发展推进机制的意见》、《太原市经济社会发展综合考核评价机制》等推动科学发展的十个体制机制创新文件。申报国家循环经济标准化试点市取得重要进展。

重点领域改革继续深化。国企改革三年攻坚阶段性目标基本完成，三年来累计筹集改革成本34.5亿元，5.1万职工劳动关系得到调整，1.5万职工公益性岗位就业，所有改制企业职工补交了五大保险，所有市属企业职工进入医保。政府机构改革基本完成，事业单位分类改革进展顺利。实施增值税转型改革，全面落实结构性减税政策，减轻企业税费18.6亿元。着力保障民生，支出结构进一步优化。广泛开展“银企保信投”对接服务活动。市政府与民生银行展开业务合作，各县区分别组建小额贷款公司，对中小企业的金融服务不断改进。全省首家外资银行汇丰银行入驻我市。

科技创新稳步推进。全市研发经费投入增长7.3%。颁布实施《太原市科技风险投资促进条例》、《太原市促进科技服务业发展办法》两部地方科技法规。经省鉴定的科技成果增长75%。9个项目获国家科学技术奖。荣获“全国科技进步先进市”称号。大力开展 “民生科技促进年”活动，科技成果转化率达到45%。太原技术转移促进中心成为国家技术转移示范机构，太原市高新技术创业服务中心成为国家级大学生科技创业基地试点。全市专利授权量增长23%，专利转化率40%。拥有自主知识产权的规模以上工业企业达到30.9%。顺利通过国家知识产权示范市验收。

政务环境持续改善。加强动态监管，对审批事项进行清理和对接，取消审批事项20项，依法调整50项。对市级审批事项分类集中办理，窗口承诺件提前办结率97.9%。规范窗口审批程序， 202项审批事项授权窗口办结，窗口办结率提高35个百分点。开辟“绿色通道”，确保重点项目顺利推进。围绕120多项事关全市发展全局的重点问题和群众关注的难点、热点和焦点问题，组织73次“三位一体”联合政务督查，有效推动了工作落实。

民主法制建设不断加强。自觉接受人大和政协的监督，办理人大代表建议和政协委员提案762件，答复率100%。围绕全市中心工作，认真听取各民主党派、工商联、无党派代表人士的意见。大力开展法制宣传和法律援助，全民法制观念逐步增强，“五五”普法效果明显。民族、宗教工作保持平稳。强化行政监察和审计监督，政府自身建设得到加强，廉政建设责任制全面落实。

各位代表，回顾攻坚克难、负重奋进的2009年，我市经受住了国际金融危机的严峻考验，总体上完成了“三保一促”的战略任务，成绩来之不易。这是省委、省政府和市委正确领导的结果，是市人大、市政协监督支持的结果，是全市人民团结奋斗的结果。我代表市政府，向辛勤工作在各条战线的工人、农民、知识分子和各级干部，向大力支持政府工作的人大代表、政协委员、各民主党派、工商联、无党派人士、人民团体，向关心帮助太原发展的国家、省驻并单位和驻并部队、武警官兵，向积极参与太原建设的港澳台同胞、海外侨胞和国际友人，表示崇高的敬意和衷心的感谢！

在肯定成绩的同时，我们也清醒地认识到，我市经济社会发展还面临不少困难和问题，主要是：受国际金融危机等因素的影响，规模以上工业增加值降幅较大；外需不足对我市钢材、焦炭、煤炭和金属镁等主要产业形成较大影响；现代服务业和文化产业发展质量和水平还不高；由于经济和政策性双重因素影响，财政增收困难；城中村改造、节能减排、环境保护任务艰巨，冬季采暖期市区空气污染明显加重；体制机制创新深化不够，发展环境有待进一步改善，一些部门办事效率不高。这些问题，在国际金融危机影响下形成“叠加效应”，制约着我市加快科学发展的步伐。对此，我们将积极采取措施，认真研究解决。

二、2010年政府工作主要任务

2010年政府工作的指导思想是：以邓小平理论和“三个代表”重要思想为指导，深入贯彻落实科学发展观，围绕建设新型工业基地、特色文化名城和现代宜居城市的目标，以加快转变经济发展方式为主线，深入实施创新型城市建设主战略，整体推进绿色转型，着力加快结构调整和八大功能区建设，着力加快城乡统筹发展和民生事业发展，着力加快自主创新和改革开放，着力加快文化创新和精神文明建设，在建设太原经济圈和 “服务全省、影响全国、吸引世界”的省会城市上实现新突破。

主要预期目标是：地区生产总值增长8%以上；规模以上工业增加值增长10%；全社会固定资产投资增长20%；社会消费品零售总额增长16%；地区外贸进出口总额增长8%；一般预算收入增长8%；城镇居民人均可支配收入和农民人均纯收入均增长9%；居民消费价格总水平涨幅控制在3%左右。以上为导向性指标。

城镇新增就业人数9.5万人，城镇登记失业率控制在4%以内。万元地区生产总值能耗下降6%以上，二氧化硫和化学需氧量排放量完成省政府下达的减排任务。以上为约束性指标，必须完成。

根据上述指导思想和预期目标，今年政府工作要以“调结构、促转型、稳增长、保民生”为重点，全面完成“十一五”规划任务，突出做好以下七个方面的工作：

（一）加快发展绿色产业，在产业结构升级转型上迈出新步伐。

着力实施“绿色百强”工程。以绿色产业为主攻方向，大力调整工业结构，加快工业绿色转型的步伐。重点发展装备制造、现代煤化工、新型材料、食品加工等新兴产业和新能源、电子信息、生物制药、节能环保等高新技术产业，全力推进七大产业集群发展。设立绿色转型引导基金，着重抓好210个重大技术改造项目，力争用三到五年时间，培育100个左右产值10亿元至100亿元以上的“绿色百强”企业，形成新的增长点与增长极。各县区也要积极培育“小一号”的绿色企业群，加快县域经济发展。

大力发展都市现代农业。以工业化、产业化、标准化提升农业，加强农业基础设施建设，着力推进全省农业新品种、新技术科技示范中心建设，着力推进高效农业、精品农业、生态农业和休闲观光农业展示中心建设，着力推进绿色安全农产品加工中心、农产品物流中心建设，积极发展创意农业，加快农业结构性调整。推进农业标准化生产和无公害蔬菜生产基地建设。建立健全农产品质量安全检测检验体系、重大动植物疫病防控体系和农业科技服务体系。农业新技术覆盖率达到95%以上。大力发展农业产业化经营，以建设农产品深加工强市为目标，抓好五大加工产业，延伸产业链条，做强龙头企业，提升产品竞争力。

保持服务业强劲发展势头。加快发展绿色服务业，在电子商务、物流配送、文化创意等18个服务业重点领域，支持旗舰企业和重点项目加快发展，形成具有较强带动作用的绿色服务业企业群。各城区要大力培育一批绿色骨干服务业企业，形成新的增长点。实施文化产业振兴工程，抓好文化产业园区和文化基地建设，引导重大文化产业项目向基地和产业园区集中。扶持民营资本发展文化产业，培育一批竞争力强的骨干文化企业。编制完成《太原市旅游产业发展总体规划（2010-2020）》。开展“旅游服务质量提升年”活动。继续实施旅游“一卡通”惠民工程，推出四省六市旅游“一证通”。旅游总收入增长15%。

推动园区经济上规模、上层次。各经济园区要当好全市转变经济发展方式的“火车头”。高新区要以建设创新型园区为中心，全力推进国家科技兴贸创新基地和国家火炬计划软件产业基地建设，加速壮大煤化工技术研发、IT、文化创意、新材料新能源和环保节能五大特色产业集群。经济区要积极创建国家级生态工业园区，加快推进煤机装备国家新型工业化产业示范基地建设，围绕富士康、长安重汽、太重煤机、智奇高速轮对等龙头企业完善产业配套，促进产业集群的形成。民营区要着力构建现代物流核心平台和新型工业发展平台，实施国家物流服务标准化试点，加快培育物流龙头和工业骨干，建好汇大物流、横店工业园等项目。不锈钢园区要大力推进国家新型工业化产业示范基地不锈钢深加工基地建设，做好省循环经济试点工作。

抓紧节能降耗和节水工作。继续推进煤炭资源整合、煤矿兼并重组，重点抓好煤矿复工复产、升级改造、安全监管、小煤矿关闭等工作，全面提升煤炭产业素质。大力发展循环经济，推进节能科技进步，推广低碳技术。抓好太钢、西山、太化、美锦等两批循环经济试点企业的项目实施。做好煤层气、煤矸石、地热等资源的综合利用工作。推进燃煤锅炉改造、余能利用、绿色照明、天然气置换、垃圾分类处理和资源化利用等节能项目建设。加强建筑节能和可再生能源的推广应用。年综合能源消费总量超过1000吨标煤的固定资产投资新开项目，必须进行合理用能评估。深入开展高耗能行业能效对标活动，严格节能执法检查，全面开展强制性节能审计。推进节水型社会建设，继续实施关井压采，加大污水处理和中水回用力度，加强节约用水宣传教育和制度化管理。实施严格的耕地和矿产资源保护制度。

（二）全面展开重大项目群建设，在优化经济布局、拓展发展空间上实现新突破。

推进八大功能区建设。编制完成八大功能区总体规划。制定产业扶持政策，在建设规划、土地征用等方面优先安排，在投融资、行政收费等方面给予优惠。在优化结构的前提下，坚持基础设施投资与产业开发投资并重，进一步扩大投资规模，突出抓好96个在建和新建项目。各县区要围绕八大功能区发展目标，在环境治理、资源整合、企业搬迁、园区建设、产业发展、企业集聚等方面实现突破。

加快西山地区综合治理。用足用好西山地区综合整治计划单列政策，出台西山地区综合整治实施条例，理顺依法整治运作机制。加快长风西大街两侧8.4平方公里启动区的建设。继续实施以植树造林和河湖治理为主要内容的生态修复工程，完成义务植树1万亩，开展“一湖六河”治理等14项生态建设工程。大力推进企业搬迁、关停淘汰工作，关停一批小型区属污染企业，涉煤、建材、焦化三类企业在三年内基本退出西山整治区。扶持铁路装备工业园、晋西春雷铜材等新型产业建设项目加快发展。切实做好区内重点文物保护和恢复性开发建设工作，抓好西山旅游循环公路、蒙山二期等7项文化旅游项目。

积极推动太原经济圈建设。编制完成综合交通运输规划，构建太原经济圈综合交通体系和路网框架。完成太原环城高速公路太谷一级公路连接线太原段的建设任务，做好太古高速、太佳高速、阳平高速、大西铁路、太中银铁路等国家和省重点建设工程的协调服务。抓好太原铁路枢纽西南环线等项目建设，加快汾东新区建设，继续扎实推进太榆同城化进程。

努力破解土地和资金紧缺难题。认真抓好项目选择、资金筹集和建设用地落实等项目前期工作。多渠道争取用地指标。加大盘活存量土地力度，挖掘现有建设用地潜力。推进城乡建设用地增减挂钩和集体建设用地流转，拓展用地空间。推进差别化土地计划管理制度，优先保证重点建设区域、中央和省扩大内需项目、重大基础设施、重点产业、民生以及承接产业转移项目用地。实行节约、集约用地考核评价制度，提高土地利用效率。全力做好融资工作，积极争取国债和省级各部门安排的建设资金。落实支持社会投资的各项政策措施，保护好多种投资主体的积极性，引导从煤炭行业退出的民间资本转型发展，扩大民间投融资规模。完善市级投融资平台，加强金融服务，确保配套资金及时到位。

各位代表，加快构建绿色产业体系，拓展经济发展空间，是加快转变经济发展方式、推进科学发展的战略选择，事关太原未来发展大局。我们一定要担当起历史赋予的神圣使命，扑下身子、埋头苦干，在新一轮产业革命和绿色经济浪潮中，创造无愧于人民、无愧于历史的新业绩！

（三）在关键领域和薄弱环节狠下功夫，确保经济平稳较快增长。

支持非公有制经济快速发展。围绕全市产业布局，促进非公有制经济快速成长。全面落实促进民营经济发展的政策，放宽民间资本的市场准入条件。健全重点企业预警机制，加强信息沟通，开展“送信心、送政策、送服务”活动。发挥商会在区域联系和行业发展中的积极作用。发展为中小企业融资提供担保的政策性、商业性、互助式会员制担保机构，搭建中小企业融资平台。规范发展小额贷款公司。引导企业推进私募股权、上市等多元化融资，多渠道解决企业融资难问题。

促进工业经济稳步上升。加大对工业的资金、政策扶持力度，着力解决制约工业经济稳定增长的深层次问题和薄弱环节，妥善处理亏损企业生产经营中遇到的实际问题。引导企业狠抓内部管理，开拓市场，帮助企业走出困境，加快发展。

进一步扩大内需拉动消费。加快农村市场流通网络建设，继续抓好“家电下乡”、“汽车下乡”。大力实施万村千乡市场工程，在经营网络空白村新建一批农家店和配送中心。升级改造和新建10个标准化集贸市场。推进供销社农产品直销和农副产品购销体系建设。积极培育消费热点，拉动节假日和会展消费，促进宽带网络等信息消费。实施“家政服务工程”。对尖草坪小商品批零市场进行升级改造。支持社区商贸、便民服务、废旧物资回收等传统服务业加快发展、升级转型。

努力促进对外贸易回升好转。优化外贸进出口结构，加快出口产品结构调整和技术升级，引导开发具有自主知识产权的出口产品。实施名牌战略，提高产品的科技含量和附加值，增强在国际市场的竞争力。积极开拓新兴市场和发展中国家市场，扩大对巴西、印度、东盟等市场的出口，弥补对欧美市场出口额的减少。强化外贸企业防范风险的意识，保障出口收汇安全。

确保城乡居民收入稳定增长。深化收入分配制度改革，逐步提高劳动报酬在初次分配中的比重。严格落实最低工资制度。积极帮助和推动农村富余劳动力外出打工，拓展农民创业途径。完善农村科技服务体系，发展农民专业合作社，带动农民增收致富。改善农村金融服务。加大对养殖、种植大户的财政扶持力度。继续抓好扶贫开发、整村推进、老区建设等工作，确保贫困地区农民稳定增收。

（四）加强城乡规划、建设和管理，推进现代宜居城市和新农村建设。

加快城市规划和基础建设步伐。完成城市总体规划、历史文化名城保护规划、轨道交通线网规划等重点规划的报批工作，做好西山地区综合整治、南客站及省市十大重点工程的规划编制、审批及协调工作，编制完成静态交通、防震减灾等20项专项规划。加快150项重点工程建设。突出抓好续建工程和南客站、长风商务区两大片区建设工程。在老城区外围建设一批城市道路，改造中心城区道路，提高主干线通行能力。年内开工建设轨道交通工程。抓好19个道桥项目和9个防洪排水项目，高标准建设滨河东路北延、城南污水处理厂等项目。加强对“城中村”的管理，加快整体改造步伐。扩大保障性住房建设规模，棚户区改造354万平方米，经济适用房建设183万平方米，配建廉租房18万平方米。按照“依法、共赢、和谐、稳定”的要求，稳妥做好各类建设项目的拆迁安置工作。

扎实推进新农村建设。认真实施《太原市绿色村庄要求与评价》等绿色标准，结合创建“省级新农村建设示范村”、“市级新农村建设绿色转型示范村及标杆村”，以“五个全覆盖”为重点推进新农村建设。高标准推进“四化四改”和“五个一”工程。积极推广使用生物质能源、太阳能照明等新能源，重点发展大中型沼气、秸秆集中供气工程。加强农田水利基本建设，改善农村用水条件。加强水土保持生态治理，抓好淤地坝建设。完善农村路网结构，全面完成开行贷款县乡公路改造和建设任务，“村村通”水泥（油）路工程向自然村延伸，维修改造损坏严重的村通水泥（油）路。

加强生态建设和环境保护。实施生态强市战略。完成造林26.89万亩，育苗2.5万亩。加快推进集体林权制度改革。积极建立生态补偿机制，启动碳汇造林项目，支持娄烦做好省城水源地保护工作，加快阳曲东西山生态建设，抓好古交“树木年度直补、商品化兴林”试点工作。巩固国家园林城市创建成果，启动国家生态园林城市建设工程，抓好滨河东路北延、滨河西路南延风景林带等重点绿化工程，绿化覆盖率、绿地率均提高1个百分点，人均公共绿地面积增加0.5平方米。加强地质灾害治理，做好西山国家矿山公园、虎峪沟神底沟等地质环境治理项目的申报工作。以创建环保模范城为载体，整体推进“减排、蓝天、碧水、生态、区域整治、环境安全”六大工程。加强环境敏感区域综合整治，继续淘汰落后产能，严格控制重点工业企业排污总量。完成河西北中部污水处理厂改造等污水处理工程，在电力、焦化、冶金等重点行业实施一批污染物减排工程。启动晋阳湖及13条边山支流的环境治理。对市区内24个城中村进行集中供热或区域供热改造，集中供热扩网1500万平方米，拆除燃煤锅炉732台，有效缓解冬季大气污染。机动车尾气参检率不低于80%，对黄色标志车辆实施限行。市区空气质量二级以上天数达到290天以上。

开创城乡管理新局面。以创建全国文明城市为标杆，实施《太原市城市市容绿色管理规范》，全面加强城乡管理与服务。狠抓环境治理，以精细化管理、长效机制建立为重点，继续引深拓展城乡清洁工程，对全市范围内的违法建设、建筑工地、便道街巷、环境卫生进行集中专项整治。注重功能提升，加强对市政基础设施、小街小巷、供水排水、供气供热、污水处理、垃圾处理、防汛排洪等设施和项目的建设、维护、改造。坚持民生优先，加强市政公用行业的运营管理，确保设施运行安全稳定，公共服务达产达标。开展道路畅通行动，解决出行难题。严格掘路审批，规范便道占用；加快无障碍设施建设；合理规划建设停车场，开发立体和地下停车场资源；大力发展公共交通，加快车辆更新和站场建设。健全治理车辆超限超载工作长效机制，高标准做好“治超”工作。推进城乡管理绿色转型，开展绿色文明街道、绿色文明工地、绿色宜居社区创建活动。突出科技引领，加快数字城管系统的硬件建设和软件升级，推进数字城管向城乡交界和县区覆盖，努力构建“数字城管、法治城管、服务城管、和谐城管”。

（五）突出做好“保民生”各项工作，加快发展社会事业，提升和谐太原建设水平。

做好就业和社会保障工作。大力推进国家级创业型城市创建工作，设立创业扶持资金，完善落实扶持政策，实施创业促就业工程。做好高校毕业生、困难群众、农民工、残疾人等就业困难群体的就业服务工作。依托太原市高级技工学校，建设太原市高技能人才公共实训基地。发挥技工院校的主渠道作用，加强对下岗失业人员、农村转移劳动力、退伍军人等群体的技能培训和创业培训，提高创业和就业能力。强化劳动保障监察，规范劳动用工行为，落实企业裁员报告制度。继续扩大各类社会保险的覆盖面。推进城乡一体化低保救助体系建设，扩大城乡困难群体医疗救助范围。加快发展慈善事业，扎实开展“慈善一日捐”等慈善救助活动。

认真解决民生热点问题。完善“物价上涨与困难家庭临时生活补贴联动机制”，保障低收入困难群体的生活质量。加强对食品和餐饮服务环节的安全监管，建立食品安全信用档案，规范小企业、小作坊的生产加工行为。深入开展药品安全、医疗器械安全、虚假违法广告、农村市场等专项整治。严厉打击传销和商标侵权行为，营造良好的市场环境。加强价格综合调控，稳步提高粮食最低收购价。完善生猪市场调控预案，合理引导市场预期。规范征地程序，依法及时足额补偿，维护农民权益。提高防灾减灾工作水平。

加快发展教育、卫生事业。继续推进“百校兴学”工程和校校有标准操场工程，进一步提高全市学校基础设施水平。10县区全部通过省义务教育标准化验收。净化校园周边环境，完善安全设施。继续做好“两免一补”工作。大力发展职业教育，制定农村学生免费接受中等职业教育的政策。全面实施教师绩效工资制度，规范学校办学行为和教师从教行为。加强教师队伍建设，提高教育教学水平和质量。完成太原大学新校区一期工程。加快医药卫生体制改革和公立医院改革试点步伐。推进农村新型合作医疗由“县办县管”向 “市办县管” 模式转变，筹资标准提高到人均150元，参合率稳定在95%以上。社区卫生服务网络100%全覆盖，农村三级卫生网络两年内实现“村覆盖、乡达标、县提高”的目标。完善重大疾病防控体系，提高预警和应急处置能力。扎实做好各类传染病的防控工作，提高防治能力。大力扶持和促进中医药事业发展。完善医疗援助长效机制，落实好对口支援、济困病房、单病种最高限价等惠民举措。

推进文化、体育等各项事业发展。扎实做好国家历史文化名城申报工作。全面推进太原美术馆、县区图书馆、文化馆、乡镇文化站、农村文化活动室等四级公共文化设施建设。繁荣文艺创作，打造文艺精品。办好晋商文化艺术周等品牌文化活动。继续开展文化下乡活动，扎实推进文化惠民工程。积极发展网络视频、移动电视等新媒体。做好非物质文化遗产保护和文物保护修复工作。加强文化市场监管和综合执法。加快滨河体育场建设。承办好山西省第十三届运动会。积极备战全国第七届城市运动会和伦敦奥运会。广泛开展全民健身运动，实施农民体育健身工程。稳定低生育水平，提高人口素质。全面开展太原市第六次全国人口普查工作。加快市档案馆建设。开展“科普进社区”活动。落实党的民族宗教政策。气象、地震、外事、侨台、地方志、民防、双拥等各项工作和老龄、妇女、残疾人、关心下一代等各项事业，都要实现新发展，取得新成绩。

切实维护社会安全稳定。以“晋阳风暴”系列严打整治行动为依托，依法严厉打击各类违法犯罪活动。加强社会治安防控体系建设，坚决遏制“两抢一盗”犯罪行为。深入推进社会矛盾化解、社会管理创新、公正廉洁执法三项重点工作。加强交通、消防安全管理，加大对酒后驾驶、火灾隐患等问题的整治力度。强化安全发展理念，全面贯彻“安全第一、预防为主、综合治理”的方针，继续深化“安全生产年”活动，巩固专项整治成果。以治理隐患、防止大事故为重点，深入开展隐患排查治理，对非法生产经营活动始终保持高压态势，严肃查处各类安全生产事故，不断提升安全生产水平。深入开展矛盾纠纷排查调处工作，继续开展市县两级“大接访”活动，依法及时解决信访群众反映的问题，实现信访形势持续明显好转。全面加强应急管理，完善应急机制，及时妥善处置各类重大突发事件。

各位代表，保障和改善民生，确保和谐稳定，是政府义不容辞的责任。我们要尽最大努力，投入更多财力和精力，多做雪中送炭的工作，不断解决人民群众关注的热点和难点问题，让人民生活得更加幸福、更有尊严，让社会更加公正、更加和谐！

（六）加快自主创新、改革开放、民主法制与精神文明建设，不断增强科学发展的动力与活力。

强化科技支撑作用。积极创建国家创新型城市试点，全社会研发经费占GDP的比重突破2.2%。大力开展“科技创新促进转型发展年”活动，在绿色转型引导基金中专设绿色科技支持资金，增强科技创新对转型发展的支撑力。围绕八大功能区建设，大力开发和应用战略性新兴产业关键技术。支持企业开展产学研战略合作，在支柱产业和特色领域培育一批产业集群技术联盟，经省鉴定的科技成果增长20%；推进科技成果产业化，科技成果转化率突破50%，高新技术产业增加值占GDP的比重超过8%。加快发展生产性科技服务业，建设公共科技信息、公共技术研发、公共技术转移等科技创新服务平台，科技服务业增加值突破60亿元。加强知识产权示范市建设，完成《太原地区科技资源开放共享条例》立法工作，专利授权量增长32%。全市科技进步指数达到26，在全省保持领先。

深化各项改革。巩固国企改革成果，完成企业破产终结和改革“回头看”工作，抓好辅业资产的整合管理。完善国有资产监管体系建设，加快国有资本调整和国有企业重组，抓好重点项目建设，发展壮大长安重汽、锅炉集团、东山煤矿等“十大项目”。深化财税体制改革，落实好结构性减税等优惠政策。调整优化支出结构，推进“五个全覆盖”，全力保障事关民生和保持社会和谐稳定的刚性支出，保证科技创新、重点建设项目资金需求。做好政府机构改革后续工作，保证政府各部门行政运转高效、职能履行明晰、内设机构合理。积极推进事业单位分类改革，落实绩效工资政策。做好乡镇机构改革的准备工作。深化文化体制改革，解放和发展文化生产力。

扩大对外开放。优化外商投资结构，引导外资投向高新技术产业、现代服务业、环保产业。鼓励外商投资传统产业的技术改造和升级，严厉禁止投资“两高一资”项目。积极组团参加国内大型招商引资经贸洽谈活动，抓好重点项目的推介。做好项目提前对接，提前落实用地、环保等条件，提高签约率。做好引资项目的审批、履约与跟踪服务，重点抓好合同外资1000万美元以上项目的审批与跟踪服务，协调解决这些项目在立项、规划、土地、环评等审批方面遇到的问题。对中博会、煤博会签约项目，及时跟踪了解履约情况，提高项目履约率和资金到位率。

编制好“十二五”规划。高起点、高质量做好太原市国民经济和社会发展第十二个五年规划纲要的编制工作。要综合考虑国内外经济社会发展的新情况、新特点，围绕加快转变经济发展方式、关注和改善民生等重大问题，突出绿色经济、循环经济、低碳技术要求，强化理念创新，强化衔接协调，强化调查研究，强化民主参与，提高规划的前瞻性、科学性、可操作性，充分体现率先科学发展、构建和谐社会、建设新太原的战略要求。

加强民主法制和精神文明建设。加快推进法治太原建设进程，圆满完成“五五”普法任务，提高全民法制意识。认真执行市人大及其常委会的决议和决定，广泛听取各民主党派、工商联、无党派人士的意见和建议，加强重大事项决策前的协商，认真办理人大代表的议案和政协委员的提案。严格依照法定权限和程序履行职责，自觉接受人大的法律监督、政协的民主监督和社会的舆论监督。加强社区管理，做好第四届全市社区居委会换届选举工作。加强基层民主建设，健全政务公开、企务公开、村务公开等制度，保障人民群众的知情权、参与权、表达权、监督权。以社会主义核心价值体系建设为基础，不断加强社会公德、职业道德、家庭美德、个人品德建设。深入开展文明和谐创建活动，提高公民文明素质和社会文明程度。

（七）全面加强政府自身建设，切实提高政府执行力。

严格依法行政。深入贯彻依法行政实施纲要，严格按照法定权限和程序行使职权、履行职责。进一步界定各执法部门的执法职责，坚决克服多头执法等问题。大力推行政府绩效管理和行政问责制度，加大对滥用权力、失职渎职和侵权行为的追究力度，严肃整治行政不作为和乱作为现象，提高政府公信力和执行力。建立健全重大决策调查研究、社会听证以及决策反馈纠偏机制和重大决策失误责任追究制度。

提高服务质量。加快转变政府职能，增强社会管理和公共服务意识，提高经济调节和市场监管能力。进一步优化发展环境，深化行政审批制度改革，深入清理行政事业性收费项目、涉企经营服务性收费项目和部门审批中互为前置的要件。推行相对集中许可权制度。充分发挥“12345”便民服务热线和《舆情专报》服务市民、服务决策的功能。围绕“绿色百强”工程，扩大全程代办事项范围，改进政府管理与服务方式，推进政务服务体系建设。

加强绿色治理。全面实施《太原市绿色转型促进条例》等法规，整体加快绿色转型。认真落实《建立健全绿色和循环经济发展推进机制的意见》等文件，突出抓好科学发展标准化工程，完善政府绿色管理机制，继续加快制定重点行业的绿色标准。着力实施已发布的27个绿色标准。做好循环经济标准化试点工作。健全“绿色十佳”政策激励机制和长效管理办法，深入推进“绿色十佳”创建活动。在政府机关继续加强绿色管理，在全社会倡导绿色消费。

狠抓工作落实。各级政府和领导干部要牢固树立科学的政绩观，把更多时间放在深入基层、调查研究、解决问题上。强化底线管理，完善各级干部目标责任、业绩考核、末位追究和领导包抓重点工作等管理考核办法。强化“三位一体”联合督查机制，落实重点工作定期督查通报、绩效评议、落实回告等制度，坚决问责追责、奖优罚劣。克服官僚主义和形式主义，改进会风、文风，切实减少不必要的会议、庆典、论坛和应酬。

坚持廉洁从政。全面落实政府系统党风廉政建设责任制，继续推进惩治和预防腐败体系建设。加强行政监察、审计监督和政府督查，继续推进煤焦领域反腐败专项斗争，深入开展工程建设领域突出问题的专项治理工作，继续开展商业贿赂治理和小金库治理专项工作。发扬艰苦奋斗的优良传统，坚持勤俭办一切事业，严格控制楼堂馆所建设，禁止高档装修办公楼。围绕群众反映强烈的突出问题，继续加大对卫生、教育、交通等领域不正之风的专项治理力度。

各位代表，今年市政府向全市人民承诺办好以下十件实事：一是全面完成中小学校舍安全改造加固任务，把学校建成最安全、学生家长最放心的地方。二是落实国家基本药物制度，年底前覆盖全市60%以上的县区基层医疗卫生机构。三是提高村卫生室建设管理水平，85%的村卫生室达到国家“五化”标准。四是在全市建立新型农村社会养老保险制度，投入1.1亿元对全市64.8万名农村适龄参保居民在制度上实现全覆盖；对全市60岁以上的农村老年人按月发放养老金，实现“老有所养”。五是援助1.06万名就业困难人员实现再就业；对2150名贫困残疾人实施康复救助，其中儿童260名，为260户农村贫困残疾人家庭实施危房改造，为100个社区（机构）配发康复训练器材。六是新建保障性住房132.5万平方米、14966套，全面完成城市和国有工矿棚户区年度改造任务。七是解决好13万农村人口的安全饮水问题。八是新建100家连锁农家店(便民店)，建设改造12家放心粮油配送中心，完善提升150家骨干示范店，建设完善500个经销网点。九是新（改）建235个村级组织活动场所。十是加大财政补贴，发展绿色公交，降低公交票价，改善市民出行条件。

各位代表，让我们以科学发展观为指导，按照省委、省政府的决策部署，在市委的坚强领导下，同心同德，攻坚克难，全力以赴加快转变经济发展方式，千方百计保障和改善民生，全面完成“十一五”规划，为“十二五”规划强势启动创造良好条件，为建设服务全省、影响全国、吸引世界的省会城市而努力奋斗！
